# Effects of increasing supplementation rates of extruded distillers’ grains cubes on stocker steer production grazing native range in Western Oklahoma

**DOI:** 10.1093/tas/txaf128

**Published:** 2025-09-19

**Authors:** Z Grigsby, S A Gunter, D Lalman, M New, C Worthington, P A Beck

**Affiliations:** Department of Animal and Food Sciences, Oklahoma State University, Stillwater, OK 74078, United States; USDA-Agricultural Research Service, Oklahoma and Central Plains Agricultural Research Center, El Reno, OK 7801, United States; Department of Animal and Food Sciences, Oklahoma State University, Stillwater, OK 74078, United States; Oklahoma State Cooperative Extension Service, South Central Research Station, Chickasha, OK 730185, United States; Oklahoma State University, Ag Research, Marvin Klemme Range Research Station, Bessie, OK 73622, United States; Department of Animal and Food Sciences, Oklahoma State University, Stillwater, OK 74078, United States

**Keywords:** Growing beef cattle, Native range, Nitrogen excretion, Supplementation rate

## Abstract

Our objectives were to determine the response curve of increasing supplementation rate with extruded distillers’ grains (DDG) cubes for animal performance, supplement conversion ratio, and N excretion by steers grazing midgrass prairie in western Oklahoma. A 2-yr trial was conducted at the Marvin Klemme Range Research Station (Klemme) and the USDA-ARS Southern Plains Experimental Range (SPER) in western Oklahoma. At Klemme, steers (Yr 1, *n* = 133, BW = 247 ± 24.5 kg; Yr 2, *n* = 134, BW = 264 ± 29.1 kg) were allocated to 6 pastures in Yr 1 and 7 pastures in Yr 2 which were assigned to three daily supplementation rates of DDG cubes: (i) Negative Control (NC)- no supplementation; (ii) Low supplement (LS) - 0.91 kg/steer and (iii) Medium Supplementation (MS) – 1.82 kg/steer. At Klemme, steers were stocked at 2.0 ha/steer in yr 1 and 2.8 ha/steer in yr 2. At SPER steers (*n *= 119 each year; Yr 1, BW = 295 ± 28.8 kg; Yr 2, BW = 294 ± 25.7 kg) were allocated to 12 pastures stocked at 2.0 ha/steer with pastures assigned to: (i) NC; (ii) LS; (iii) MS and (iv) High Supplement—daily supplementation rate of 2.72 kg/steer. Data were analyzed by year and experimental site as a completely randomized design. Least-squares means were separated using orthogonal polynomial contrasts. In both years at Klemme, the increased supplementation rate linearly increased (*P *≤ 0.01) average daily gain (ADG) but made no difference (*P* ≥ 0.16) in supplement conversion ratio. At SPER, ADG increased linearly (*P *< 0.01) in Yr 1, but quadratically (*P *= 0.05) in Yr 2; in both years, supplement conversion ratio displayed a quadratic response (*P *= 0.01), decreasing with increasing supplementation rate. These data suggest that supplementing extruded DDG cube to steers grazing native range is a viable option to intensify production on stocker operations. If forage availability and nutritive quality is adequate to support BW gains higher supplementation rates are likely not economically efficient, however if forage is limiting improved performance may be adequate for positive economic returns. The response curves observed in this research will allow producers to make supplementation decisions based on market and range conditions.

## Teaser

Growing stocker steers grazing native range can benefit from increasing supplementation rates when forage conditions limit their production. This research provides a biological basis for producers to make supplementation decisions based on pasture conditions and market circumstances.

## Introduction

Supplementation is a management practice that can be utilized to intensify production of grazing steers. Supplemental protein for growing calves consuming low-quality forage-based diets increases intake and digestibility of the forage due to correction of a deficiency in rumen degradable protein (Rawson et al. 2017; Cox and Wickersham 2023). However, in cases where forage allowance is limiting, increased forage intake may not be a favorable outcome. Instead, partial substitution of forage intake with supplements without negatively influencing BW gain could be a more desirable result. Substitution of forage intake by supplementation has been found to range between 0.3 and 1.1 kg of forage for each additional kg supplemented (Morris et al. 2005; Adams et al. 2022b; Cazzuli et al. 2023b). Although increased supplementation rates can positively influence animal performance, supplemental conversion ratio (kg added gain/kg supplement) often suffers (McCollum and Horn 1990; Bodine and Purvis 2003). This decrease in supplement conversion ratio is often most prevalent when forage protein is adequate (Moore et al. 1999; Womack et al. 2024) and performance response to supplementation is predominantly from increased energy supply. Increasing the ability to efficiently utilize potentially limited forage is possible with DDG supplementation. The cattle market often plays a role in management decisions such as the decision to more highly value increased bodyweight (BW) gain versus the efficiency of BW gain. A stable extruded DDG cube has become available that provides advantages for pasture supplementation that reduces the risk of product loss due to wind and soil mixing as seen with loose DDG. Adams et al. (2022b) found that the extruded DDG cubes had greater contents of fat, neutral detergent insoluble crude protein, and total digestible nutrients, but lower (*P* ≤ 0.01) neutral and acid detergent fiber than loose DDG. Our goal was to provide beef producers with management programs that can increase both animal productivity and economic sustainability without negative implications on the rangeland ecosystem. Our current research was to investigate the impacts of increasing supplementation rates of DDG cubes and the relationship between animal growth performance and supplement conversion ratio for growing steers grazing native rangelands in the midgrass prairie ecosystem in two locations in western Oklahoma.

## Materials and methods

All animals and procedures used in this experiment were approved by the Oklahoma State University Institutional Care and Use Committee (IACUC protocol # AG-22-16) and approved by USDA-ARS Southern Plains Range Research Station IACUC (Protocol # ACUP-027).

### Experiment 1. Marvin klemme range research station (klemme)

A 2-yr supplementation rate trial was conducted at the Marvin Klemme Range Research Station (Klemme), near Bessie, Oklahoma (35°25′00.4″ N, 99°03′42.6″ W). Precipitation data for the study was collected at an onsite weather station (Oklahoma Mesonet 2024).

#### Treatments

At the Klemme Range Research Station, we imposed 3 treatments with incrementally increased supplementation rates: (i) Negative Control (NC) – No supplementation except *ad libitum* access to complete mineral mixture; (ii) Light Supplementation (LS)—daily supplementation with 0.91 kg of DDG cubes (Masterhand Milling, Lexington, NE)/steer; and (iii) Medium Supplementation (MS)—S daily supplementation with 1.81 kg of DDG cubes/steer.

##### Study site

This site consists primarily of rolling Red Shale uplands (2% to 15% slopes) dissected by deep drainages with Cordell silty clay loam soils, which are shallow (25 to 36 cm) and contain numerous rocky outcrops of hard red siltstone. These Red Shale sites support mixed-grass prairie as the potential climax natural vegetation (Gillen et al. 2000). Sideoats grama (*Bouteloua curtipendula*), blue grama (*Bouteloua* gracilis), red threeawn (*Aristida longiseta*), buffalograss (*Buchloe dactyloides*), silver bluestem (*Bothriochloa saccharoides*), purple threeawn (*Aristida purpurea*) and little bluestem (*Schizachyrium scoparium*) were the major grass species at this site in 1990 identified by Gillen et al. (2000). The study area was divided into 32 to 57-ha pastures which were allocated to the 3 treatments described above. Average annual rainfall for Klemme is approximately 72 cm with summer daytime temperatures ranging from 27° C in May to 35 °C in July (Oklahoma Mesonet 2020). Grazing took place from May 21 to October 13 (145 d) and May 13 to September 26 (136 d) in 2021 and 2022, respectively.

#### Animal management

Each year at Klemme, steers. (*n* = 133 crossbred steers, BW = 247 ± 24.5 kg and *n* = 134 crossbred steers, BW = 263 ± 29.1 kg in 2021 and 2022, respectively) were randomly allocated to *n* = 6 pastures in 2021 and *n* = 7 pastures in 2022. Stocking rates were 2.02 ha/steer in 2021 and 2.83 ha/steer in 2022. The additional pasture in 2022 was allocated to the LS treatment to accommodate reduced stocking rates due to drought conditions in 2022 due to concerns with potential decline in forage biomass availability and nutritive value. Pastures were allocated to the 3 incremental supplementation rates (*n* = 2 pastures/treatment). Steers in all pastures were provided *ad libitum* access to a complete mineral mixture (Stocker Mineral B-1440, Masterhand Milling, Lexington, NE) designed to provide 1587 mg lasalocid per kg of finished mineral mixture. All steers were implanted with 40 mg of trenbolone acetate and 8 mg estradiol and 29 mg tylosin tartrate (Component TE-G, Elanco Animal Health, Greenfield, IN) at the start of the grazing season.

Steer BW were recorded without an overnight shrink on 2 consecutive days at the beginning and end of the grazing season and on a single day in the middle of the summer. grazing period in July. The BW collected on 2 consecutive days at the beginning and end of the grazing season were averaged for estimating initial and final BW. At the beginning of the grazing season each year, steers were transported from their receiving pens at the Klemme Range headquarters and held in a dry lot trap overnight with access to water and long-stem wheat (*Triticum aestivum*) hay. Steers were gathered on d -1 at 0600 individually weighed, assigned individual identification, and then returned to the dry lot trap with access to free-choice water and hay. The BW collected on d -1 was used to assign steers to pasture groups so that each group had an equal average BW for stocking rate distribution. The groups were assigned to pastures and pastures randomly assigned to supplementation treatment. The following day (d 0) steers were gathered, weighed, sorted into assigned groups, and transported to their respective pasture. For the mid-summer intermediate BW data collection, to limit heat stress imposed on the steers, the steers were gathered beginning at first light (0600) and penned at the Klemme Range headquarters cattle processing facilities. allowed overnight *ad libitum* access to water and long-stem wheat (*Triticum aestivum*) hay overnight before individually BW collection and return to their respective study pastures beginning at 0600 the following morning. At the end of the summer grazing season, steers were gathered from their pastures beginning at 0700 and individually weighed before returning to the drylot trap overnight with access to *ad libitum* water and long-stem wheat hay. Steers were reweighed the following day beginning at 0700.

In Yr 1, one steer from the NC treatment was excluded from the analysis due to complications from chronic respiratory disease resulting in weight loss. In Yr 2, 5 steers were removed from the analysis for a variety of reasons. Two of the steers were removed from the analysis (1 NC and 1 MS) for complications from chronic respiratory disease resulting in death, one steer from LS was removed for complications from chronic respiratory disease resulting in BW loss, and two steers (1 MS and 1 LS) were removed from the analysis due to unresolved foot rot resulting in lameness and removal from treatment pastures.

### Experiment 2 USDA-ARS Southern plains experimental range (SPER)

A similar 2-yr trial was conducted at the USDA ARS Southern Plains Experimental Range (SPER) near Fort Supply, OK (36.62105°N, 99.59695° W). The SPER is located in Harper County in Northwest Oklahoma. Precipitation data for the study was collected at a weather station located 27 km from the study site (Oklahoma Mesonet 2024).

#### Treatments

At the SPER, we imposed treatments with incrementally increased supplementation rates consisting of: NC, LS, and MS described previously with an additional Heavy Supplementation (**HS**) treatment where 2.72 kg DDG cubes/steer was fed daily. Steers in all pastures were provided access to free choice range mineral (Rangeland Pro Wheat Pasture Mineral B1440 Medicated, Purina Animal Nutrition, LLC. Arden Hills, MN) designed to provide 1587 mg of lasalocid per kg of finished mineral mixture. The mineral mixture was delivered weekly to provide access of 113.5 g/d of mineral per steer.

##### Study site

Annual average precipitation at this location is 627 mm, approximately 70% coming between the months of April to September (Gunter 2019). Temperatures range from a mean of 2.3 °C in January to 28 °C in July. The Southern Plains Experimental Range site is characterized by rolling stabilized sand dunes interspersed with areas of heavier textured soils with native mixed grass prairie comprising a majority of the forage biomass. The prominent range species consist of tall, mid, and short season grasses and forbs; including sand bluestem (*Andropogon hallii*), little bluestem (*Schizachyrium scoparium*), sand dropseed (*Sporobolus cryptandrus*), blue grama (*Bouteloua gracilis*), and sand sagebrush (*Artemisia fiifolia*) (Küchler 1964; Gunter et al. 2012). Grazing took place from May 18 to September 24 (129 d) and May 20 to September 20 (123 d) in 2021 and 2022, respectively.

#### Animals management

Each year at SPER, steers (*n* = 119, BW 297 ± 29 and 295 ± 26 kg for Yr 1 and 2, respectively) were randomly allocated to *n* = 12 pastures, stocked at 2.02 ha/steer. Pastures (*n* = 12) were randomly allocated to the 4 incremental supplementation rates (*n* = 3 pastures per treatment). All steers were implanted using 40 mg of trenbolone acetate and 8 mg of estradiol, and 29 mg of tylosin tartrate (Component TE-G, Elanco, Greenfield, Indiana, United States) before turnout on pasture.

Steer BW were collected on 3 separate dates following a 16-h overnight shrink. Steers were gathered and held in drylot pens the day prior to weight collection beginning at 1600 with no access to water, feed, or forage. Shrunk BW were collected the following day beginning at 0800. All steers were sorted into their pasture groups and then trailered back to their respective pastures after BW collections were completed.

### Forage sampling and analysis

At both Klemme and SPER each year, forage biomass availability and nutritive value were analyzed by clipping 0.09 m^2^ quadrates at 2.5 cm from the ground in 10 random locations in each pasture at the beginning of the summer grazing season, at the mid-summer BW collection, and at the end of summer grazing. Forage samples were dried for 48 h at 50 °C in a forced air-drying oven. Samples were then weighed to estimate forage dry matter (DM) biomass per quadrate in order to calculate total biomass availability (kg DM/ha) for each pasture. It is well established that grazing livestock selects higher quality diets from a sward than the average of the total biomass ­(Gregorini et al. 2011), therefore leaf material was separated from stems in an attempt to mimic the forage selection. The leaf material was ground to pass through a 1 mm screen (Fritsch, Pulverisette 19, Pittsboro, North Carolina, United States) and composited within pasture for each sampling date. Following grinding, the chemical composition of leaves was estimated via near infrared reflectance spectroscopy (NIRS; NIRSD2500 F, Foss Analytics, Eden Prairie, Minnesota, United States).

### Blood urea nitrogen and N excretion calculations

Blood samples from each steer were collected at each BW collection at SPER via jugular venipuncture (6 mL, Ideal Blood Collection Tubes, Lavender Top, NEOGEN, Lansing, Michigan, United States) to later be analyzed for blood urea N (BUN) concentration. After sampling, blood samples were stored on ice during sampling for up to 3 h before transportation 30 km to the Forages and Livestock Nutrition Laboratory at USDA-ARS Southern Plain Range Research Station in Woodward, OK. Within 3 h of arrival at the laboratory, blood serum was separated by centrifugation at 3000 x g for 20 min, serum aspirated, and stored in a −20 °C freezer for later analysis at the Oklahoma State University Ruminant Nutrition Laboratory in Stillwater, OK. Serum was analyzed for urea-N using automated colorimetric procedures (Marsh et al. 1965). Estimated urinary and fecal N output, as well as estimated N intake were calculated based on steer performance BUN, and calculated N balance data from Kohn et al. (2005) using equations presented in Table 1.

**Table 1.** Equations for predicting nitrogen intake and excretion from Kohn et al. (2005).

Prediction	Equation^a^
**Urinary N, g/d**	CR x BUN x BW
**N Intake, g/d**	(UN + retained N + MFN × BW^0.75^)/TD
**Fecal N, g/d**	NI—UN—growth N

a CR = N clearance rate (1.2 L/d/kg BW), BUN = blood urea nitrogen, g/L; BW = mean BW for period, kg; retained N = 26 g N × ADG for period; MFN—metabolic fecal N = 0.04 g/kg metabolic BW; BW^0.75^ = metabolic BW, kg; TD = true protein digestibility = 0.76 kg/kg DMI; NI = N intake, g/d; and UN = urinary N excretion, g/d.

### Statistical analysis

All data were analyzed by year for each location, growth performance and BUN and calculated N balance data were analyzed using PROC Mixed in SAS 9.4 (SAS Inst. Inc; Cary, North Carolina, United States) as a completely randomized design. Because supplementation rates were uniformly increased, orthogonal polynomial contrasts were used to identify trends in the relationship between animal performance and supplementation rate. This model included supplementation rate as the fixed effect and pasture within treatment as the random effect. The analysis of forage biomass and nutritive value data were conducted using mixed procedure in SAS 9.4 (SAS Inst. Inc; Cary, North Carolina, United States). In the model supplementation rate, sampling date and their interaction were considered fixed effects and pasture within treatment as the random effect. Differences among treatments least-squares means were considered significant at *P *≤ 0.05.

## Results

### Precipitation

#### Klemme range

**Fig. 1.**
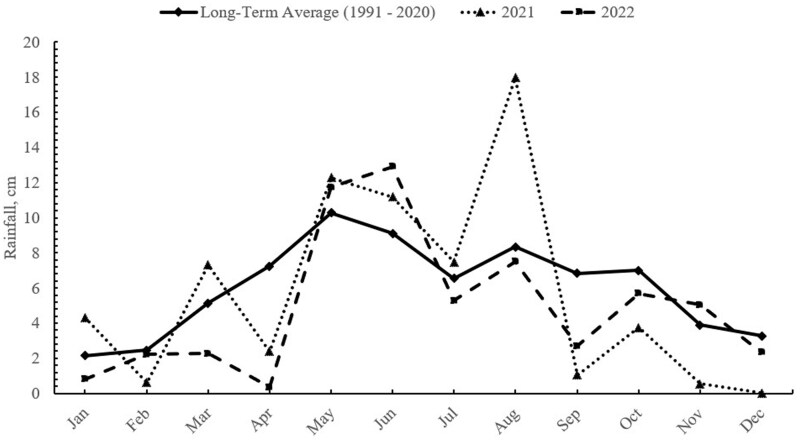
Annual precipitation at the Oklahoma state university marvin klemme range research station during the study Yr 1 (2021) and 2 (2022) compared with the long-term average from 1991 to 2020.

Monthly precipitation from January to December at Klemme for 2021 and 2022 are shown in Fig. 1. From January through September the total precipitation accumulated was 64.59 cm in 2021 which is 111% of the 30-yr long-term average from 1991 to 2020 (Oklahoma Mesonet 2024). In 2021 rainfall was generally even throughout the summer with precipitation in May, June and August being above the 30-yr average. The months from September through December in 2021 accumulated near or below the long-term average, with there being negligible precipitation in the late fall following removal of the steers in Yr 1 setting the stage for a challenging grazing season in 2022. From January through April in 2022 precipitation accumulation was only 45.75 cm, which is 79% of the long-term average. Because of the dry conditions leading up to the 2022 grazing season stocking rates were adjusted by 40% from 2 ha per steer in 2021 to 2.8 ha per steer in 2022. Precipitation during the summer growing season was near the long-term average after the start of grazing in Yr 2 (Fig. 1) promoting forage growth and providing ground water in reservoirs for livestock watering.

#### Southern plains experimental range

**Fig. 2.**
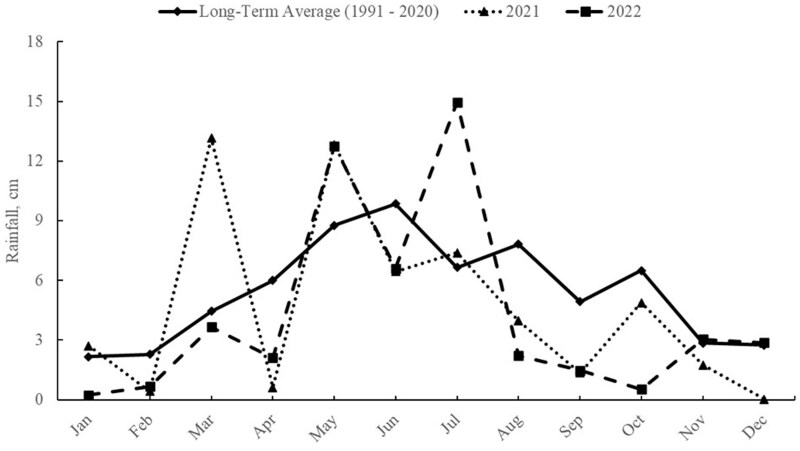
Annual precipitation at the USDA-ARS Southern plains experimental range during study Yr 1 (2021) and 2 (2022) compared with the long-term average from 1991 to 2020.

Monthly precipitation from January to December at SPER for 2021 and 2022 are shown in Fig. 2. Precipitation from January to September was 48.90 cm and 44.65 cm in 2021 and 2022, respectively which is 92% and 84% of the 30-yr average rainfall from 1991-2020 (Oklahoma Mesonet 2024). Unlike Klemme, in 2021, most of the precipitation accumulated in the early grazing season with a steady decline as the season progressed. However, in 2022 the largest proportion of precipitation was during midsummer with the remaining months being near 30-yr averages (Fig. 2). The precipitation events in the mid and late summer reinvigorated forage growth during the late summer months, enabling steers to graze fresh forage regrowth during the late summer months.

### Forage biomass and nutritive quality

#### Klemme range

Forage nutritive value and biomass are reported by year in Table 2. In both Yr 1 and 2 forage analysis showed no effect of supplementation treatment (*P *≥ 0.20) or treatment by date interactions (*P *≥ 0.12), however, sampling date (*P *≤ 0.04) had a significant main effect on forage biomass and nutritive value. In both Yr 1 and 2 forage biomass was greatest in midsummer then decreased through the end of the summer. Crude protein decreased throughout the summer while IVDMD decreased while detergent fiber concentrations (ADF and NDF) increased from the early summer into the midsummer in Yr 1 or through the late summer in Yr 2. The ratio of IVDMD: CP ranged from 9.8 to 10.5 in both years of the experiment.

**Table 2.** Forage biomass and nutritive value at the marvin klemme range research station during Yr 1 (2021) and 2 (2022).

	Sampling dates^1^				*P*-values		
Item	1	2	3	SE	Trt	Date	Trt*Date
**———————————————————————————————Yr 1 (2021) ———————————————————————————-**							
**CP, % of DM**	6.24^a^	5.55^b^	5.84^b^	0.130	0.73	< 0.01	0.94
**ADF, % of DM**	46.0^c^	46.8^b^	50.2^a^	1.10	0.49	0.01	0.46
**NDF, % of DM**	76.5^c^	79.0^b^	82.3^a^	0.18	0.20	< 0.01	0.67
**IVDMD, % of DM**	61.2^a^	58.4^b^	58.4^b^	0.17	0.98	< 0.01	0.96
**IVDMD: CP**	9.8^b^	10.5^a^	10.0^b^	0.22	0.68	0.04	0.94
**Biomass, kg DM/ha**	3071^b^	3306^a^	2827^c^	28.8	0.92	< 0.01	0.96
**———————————————————————————————Yr 2 (2022) ———————————————————————————-**							
**CP, % of DM**	6.09^a^	5.86^b^	5.76^c^	0.022	0.43	< 0.01	0.51
**ADF, % of DM**	49.7^c^	51.9^b^	55.5^a^	0.100	0.61	< 0.01	0.42
**NDF, % of DM**	76.1^c^	78.6^b^	81.8^a^	0.208	0.65	< 0.01	0.13
**IVDMD, % of DM**	61.71^a^	58.62^b^	57.17^c^	0.058	0.24	< 0.01	0.12
**IVDMD: CP**	9.8^b^	10.5^a^	10.0^b^	0.18	0.71	0.01	0.93
**Biomass, kg DM/ha**	2456^b^	2689^a^	2218^c^	16.6	0.74	< 0.01	0.72

Least-squares means within the same row with different superscripts differ *P ≤ 0.05*.

1 Sampling dates for Yr 1 (2021) were: 1—May 20, 2021; 2—July 27, 2021; and 3—October 14, 2021. Sampling dates for Yr 2 (2022) were: 1—May 19, 2022; 2—July 21,2022; and 3—September 29, 2022.

#### Southern plains experimental range

Forage biomass availability and nutritive value are reported by year in Table 3. There were no effects (*P *≥ 0.07) of supplementation or treatment by date interactions in Yr 1 or Yr 2. All measured variables showed a main effect of date (*P ≤ *0.02) with generally decreasing CP from early to midsummer and decreasing IVDMD and forage biomass with increasing detergent fiber concentrations (ADF and NDF) from the early summer through the late summer in both years. The ratio of IVDMD: CP ranged from 6.3 to 7.7 in Yr 1 and from 9.4 to 10.9 in Yr 2 of the experiment.

**Table 3.** Forage biomass and nutritive value at the USDA-ARS Southern plains experimental range during Yr 1 (2021) and 2 (2022).

	Sampling dates^1^				*P*-values		
Item	1	2	3	SE	Trt	Date	Trt*Date
**——————————————————————————————Yr 1 (2021) ————————————————————————————-**							
**CP, % of DM**	10.03^a^	7.88^c^	8.25^b^	0.052	0.75	< 0.01	0.20
**ADF, % of DM**	45.5^c^	47.0^b^	50.2^a^	0.89	0.69	< 0.01	0.43
**NDF, % of DM**	76.4^c^	79.3^b^	81.2^a^	0.19	0.97	< 0.01	0.53
**IVDMD, % of DM**	63.1^a^	60.3^b^	57.9^c^	0.13	0.07	0.02	0.08
**IVDMD: CP**	6.3^c^	7.7^a^	7.0^b^	0.19	0.98	< 0.01	0.87
**Biomass, kg DM/ha**	3372^a^	2981^b^	2787^c^	27.7	0.25	< 0.01	0.12
**——————————————————————————————Yr 2 (2022) ————————————————————————————-**							
**CP, % of DM**	6.96^b^	5.86^a^	5.80^a^	0.138	0.11	< 0.01	0.80
**ADF, % of DM**	50^c^	52^b^	54^a^	0.14	0.46	< 0.01	0.99
**NDF, % of DM**	77^c^	80^b^	82^a^	0.10	0.75	< 0.01	0.21
**IVDMD, % of DM**	65.2^a^	61.9^b^	63.1^b^	1.054	0.32	0.01	0.14
**IVDMD: CP**	9.4^b^	10.6^a^	10.9^a^	0.34	0.09	< 0.01	0.85
**Biomass, kg DM/ha**	2012^a^	1561^b^	1256^c^	756.0	0.14	< 0.01	0.88

Least-squares means within the same row with different superscripts differ *P ≤ 0.05*.

1 Sampling dates for Yr 1 (2021) were: 1—May 18, 2021; 2—July 29, 2021; and 3—September 24, 2021. Sampling dates for Yr 2 (2022) were: 1—May 20, 2022; 2—July 27, 2022; and 3—September 20, 2022.

### Animal performance

#### Klemme range

The effect of supplementation rate on performance of grazing steers for Yr 1 and Yr 2 are presented in Table 4.

**Table 4.** Effects of supplemental DDG feeding rate on performance of steers grazing mixed grass native range at marvin klemme range research station during the summer of Yr 1 (2021) and 2 (2022).

Item	Treatments^a^				Contrasts	
	NC	LS	MS	SE	Linear	Quadratic
**——————————-————Yr 1——————————————**						
**BW, kg**						
** Initial**	247	247	248	4.2	0.90	0.89
** Middle**	275	306	315	4.7	< 0.01	0.13
** Final**	327	374	387	8.3	0.01	0.18
**ADG, kg/d**						
** Early summer**	0.44	0.88	1.00	0.066	0.01	0.13
** Late summer**	0.65	0.86	0.93	0.069	0.06	0.44
** Overall**	0.55	0.87	0.96	0.058	0.01	0.20
**BW gain/hectare, kg**	40	62	69	4.1	0.01	0.20
**Supplement conversion ratio** ^b^						
** Early summer**	-	0.49	0.31	0.057	0.16	-
** Late summer**	-	0.23	0.15	0.052	0.38	-
** Overall**	-	0.35	0.22	0.049	0.21	-
**———————————————Yr 2——————————————**						
**BW, kg**						
** Initial**	265	263	264	5.2	0.85	0.79
** Middle**	319	324	330	7.4	0.34	0.98
** Final**	352	377	401	8.6	0.02	0.97
**ADG, kg/d**						
** Early summer**	0.71	0.81	0.89	0.045	0.17	0.98
** Late summer**	0.55	0.89	1.17	0.043	< 0.01	0.59
** Overall**	0.65	0.84	1.01	0.051	0.01	0.86
**BW gain/hectare, kg**	31	42	48	2.4	0.01	0.85
**Supplement conversion ratio** ^b^						
** Early summer**	-	0.09	0.07	0.064	0.83	-
** Late summer**	-	0.37	0.34	0.019	0.26	-
** Overall**	-	0.21	0.20	0.043	0.93	-

a NC-Negative Control, provided with free choice supplement only; LS-Light supplement, daily average supplementation rate of 0.91 kg/steer; MS-Medium supplement, daily average supplementation rate of 1.81 kg/steer.

b Supplement conversion ratio—kg added gain per kg supplement fed, calculated as added mean ADG in each supplemented pasture—mean ADG of NC/kg of supplement offered daily. Least-squares differences were separated using Orthogonal Contrast for supplemented treatments only.

#### Year 1

During the early summer, ADG linearly (*P *< 0.01) increased with increasing supplementation rate resulting in linear (*P *< 0.01) increases in steer BW in mid-summer BW. Although there was only a tendency (*P *= 0.06) for linear increases in ADG with increased supplementation rate in the late summer, total grazing season ADG increased linearly (*P *≤ 0.01) by 0.32 and 0.41 ± 0.058 kg with increasing supplementation rate, for LS and MS, respectively, vs NC. The increased ADG resulted in linearly (*P *< 0.01) greater BW at the end of the summer. Bodyweight gain per hectare increased linearly (*P *≤ 0.01) by 55 and 72% for LS and MS over NC, respectively. supplement conversion ratio did not differ (*P *≥ 0.16) for supplemented treatments. supplement conversion ratio in the early summer averaged 0.40 kg added BW gain/kg supplement, while, contrary to expectation supplement conversion ratio decreased to an average of 0.19 in the late summer. Over the entire summer supplement conversion ratio of LS (0.35 kg added BW gain/kg supplement) and MS (0.22 kg added BW gain/kg supplement) did not differ (*P* = 0.21) averaging 0.29 kg of additional ADG over NC per kg of supplement.

#### Year 2

Early summer ADG was not affected (*P *≥ 0.17) by the increasing supplementation rates, but late season ADG linearly improved (*P *≤ 0.01) as supplementation rate increasing from 0.55 kg for NC to 0.89 and 1.17 kg for LS and MS, respectively, compared with NC. Season-long ADG linearly increased (*P *≤ 0.01) as supplementation rate increased, from 0.65 kg for NC to 0.84 and 1.01 kg for LS and MS, respectively (*P *= 0.01) resulting in linearly (*P *= 0.02) heavier BW at the end of the grazing season with increasing supplementation rate. Bodyweight gain per hectare also increased linearly (*P *≤ 0.01) with increased supplementation. As in Yr 1, supplement conversion ratios did not differ (*P *≥ 0.26).

#### Southern plains experimental range

The effects of supplementation rate on performance of grazing steers for Yr 1 and Yr 2 at the SPER are presented in Table 5.

**Table 5.** Effects of supplemental DDG for steers grazing native range at the USDA ARS Southern plains experimental range during Yr 1 (2021) and Yr 2 (2022).

	Treatments^a^					Contrast		
Item	NC	LS	MS	HS	SE	Linear	Quadratic	Cubic
**——————————————————————————————— Yr 1 (2021)———————————————————————————**								
**BW, kg**								
** Initial**	295	297	296	295	5.4	0.97	0.84	0.99
** Middle**	344	367	371	370	5.5	0.01	0.06	0.59
** Final**	387	420	423	432	6.5	< 0.01	0.08	0.23
**ADG, kg/d**								
** Early summer**	0.68	0.98	1.03	1.03	0.059	< 0.01	0.04	0.50
** Late summer**	0.74	0.94	0.92	1.09	0.055	< 0.01	0.85	0.14
** Overall**	0.71	0.96	0.98	1.06	0.048	< 0.01	0.11	0.23
**BW gain/hectare, kg**	45	61	63	67	3.1	< 0.01	0.11	0.24
**Supplement conversion ratio** ^b^								
** Early summer**	-	0.32	0.20	0.13	0.046	0.06	0.03	-
** Late summer**	-	0.21	0.11	0.13	0.023	0.01	0.02	-
** Overall**	-	0.27	0.16	0.13	0.030	0.03	0.01	-
**———————————————————————————————Yr 2 (2022)——————————————————————————-**								
**BW, kg**								
** Initial**	297	293	295	293	4.8	0.65	0.89	0.70
** Middle**	358	384	397	403	5.6	< 0.01	0.12	0.79
** Final**	410	437	453	464	6.0	< 0.01	0.21	0.77
**ADG, kg/d**								
** Early summer**	0.90	1.33	1.49	1.61	0.048	< 0.01	0.01	0.33
** Late summer**	0.94	0.97	1.02	1.11	0.042	0.02	0.50	0.92
** Overall**	0.92	1.17	1.28	1.39	0.032	<0.01	0.05	0.37
**BW gain/hectare, kg**	56	71	78	84	1.5	< 0.01	0.05	0.37
**Supplement conversion ratio** ^b^								
** Early summer**	-	0.46	0.32	0.26	0.033	0.02	0.32	-
** Late summer**	-	0.03	0.05	0.06	0.031	0.72	0.94	-
** Overall**	-	0.27	0.20	0.17	0.005	< 0.01	0.01	-

a NC-Negative Control, provided with free choice supplement only; LS-Light Supplementation, 0.91 kg supplemental extruded DDG cubes/steer provided daily; MS-Medium Supplementation, 1.81 kg supplemental extruded DDG cubes/steer provided daily; HS-High Supplementation, 2.72 kg supplemental extruded DDG cubes/steer provided daily.

b Supplement conversion ratio—kg added gain per kg supplement fed, calculated as added mean ADG in each supplemented pasture—mean ADG of NC/kg of supplement offered daily. Least-squares differences were separated using Orthogonal Contrast for supplemented treatments only.

#### Year 1

At SPER in Yr 1, early season ADG increased in a quadratic fashion (*P *= 0.04) with a reduction in marginal response to supplementation as supplementation rate increased. In the late summer and over the entire summer grazing season ADG increased linearly (*P* ˂ 0.01) with increasing supplementation rate, which resulted in a linear increase (*P* ˂ 0.01) in BW at the end of the summer grazing season. Bodyweight gain per hectare linearly increased (*P* ˂ 0.01) with increasing supplementation rates. supplement conversion ratio during the early summer decreased in a linear fashion linearly (*P *= 0.03) and late summer supplement conversion ratio decreased in a quadratic fashion (*P *= 0.02) with increasing supplementation rate. Over the entire summer grazing season, supplement conversion ratio had a quadratic decline (*P *= 0.01) as supplementation rate increased from 0.27 kg of added BW gain per kg supplement for the LS treatment to 0.13 kg of added BW gain per kg of supplement for the HS.

#### Year 2

Early summer ADG for LS, MS, and HS increased quadratically (*P *≤ 0.01) by 0.43, 0.59, and 0.71 kg over NC, respectively (Table 5). The increase in early season ADG resulted in linear increase (*P *< 0.01) in steer BW in the midsummer as supplementation rate increased. Late summer ADG increased linearly (*P *= 0.02) with increased rate of supplementation, but supplementation only increased ADG by 0.03, 0.08, and 0.17 kg for LS, MS, and HS compared with NC, respectively. Increased rate of supplementation yielded season-long ADG that quadratically increased (*P *= 0.05) with increasing supplementation rate. Steer BW at the end of the grazing season increased in a linear fashion (*P *< 0.01) by 27, 43, and 54 kg for LS, MS, and HS, respectively, compared with NC. Bodyweight gain per hectare increased quadratically (*P *= 0.05) with increased supplementation rate. supplement conversion ratio for LS, MS, and HS decreased linearly (*P *= 0.02) in the early summer with increasing supplementation rate but did not differ (*P *≥ 0.72) among supplementation rates in the late summer. Overall, in Yr 2, as supplementation rate increased there was a quadratic decrease (*P *= 0.01) in supplement conversion ratio as supplementation rate increased.

### Blood urea nitrogen and calculated N intake and excretion

#### Blood urea N

The BUN, calculated N intake and N excretion at SPER for Yr 1 and 2 are presented in Table 6. As expected, BUN at the start of the grazing season did not differ (*P *≥ 0.23) in either year. Blood urea N concentrations at mid-summer BW and at the end of the summer grazing season collection linearly increased (*P *≤ 0.05) with increasing supplementation rate in both years of the experiment.

**Table 6.** Blood urea nitrogen concentrations and calculated nitrogen output at the USDA ARS Southern plains experimental range during Yr 1 (2021) and Yr 2 (2022).

	Treatments^a^					Contrast		
Item	NC	LS	MS	HS	SE	Linear	Quadratic	Cubic
**——————————————————————————————Yr 1 (2021) —————————————————————————————**								
**Blood urea nitrogen, mg/dl**								
** Initial**	5.78	5.41	5.57	5.50	0.236	0.53	0.53	0.49
** Midsummer**	5.95	7.09	7.32	8.06	0.638	0.05	0.76	0.63
** Final**	7.33	9.25	9.43	11.84	0.496	< 0.01	0.63	0.11
**Nitrogen intake** ^b^ **, g/d**								
** Early summer**	57.4	75.4	78.7	83.0	5.42	0.01	0.24	0.54
** Late summer**	75.9	99.0	99.8	122.3	3.85	< 0.01	0.93	0.03
**Urinary nitrogen output** ^b^ **, g/d**								
** Early summer**	25.0	30.5	31.7	34.5	2.89	0.05	0.66	0.64
** Late summer**	35.5	47.4	48.4	61.6	2.64	< 0.01	0.87	0.08
**Fecal nitrogen output** ^b^ **, g/d**								
** Early summer**	16.8	21.2	22.0	23.0	1.31	0.01	0.23	0.54
** Late summer**	21.6	27.3	27.5	32.9	0.92	< 0.01	0.88	0.03
**N Utilization, %**								
** Early summer**	26.9	31.6	32.2	30.4	1.17	0.07	0.02	0.78
** Late summer**	25.5	25.3	24.3	23.1	1.09	0.13	0.67	0.86
**———————————————————————————————Yr 2 (2022) ————————————————————————————-**								
**Blood urea nitrogen, mg/dl**								
** Initial**	6.08	5.60	5.63	5.67	0.204	0.23	0.24	0.59
** Midsummer**	5.74	6.11	7.77	7.76	0.542	0.01	0.73	0.26
** Final**	7.17	7.45	8.74	9.93	0.504	< 0.01	0.39	0.63
**Nitrogen intake** ^b^ **, g/d**								
** Early summer**	56.2	69.9	81.3	81.3	3.92	< 0.01	0.12	0.61
** Late summer**	77.6	89.9	99.3	114.5	5.29	< 0.01	0.79	0.72
**Urinary nitrogen output** ^b^ **, g/d**								
** Early summer**	24.1	26.3	33.6	33.3	2.37	0.01	0.60	0.26
** Late summer**	36.4	40.5	48.0	55.7	3.01	< 0.01	0.57	0.81
**Fecal nitrogen output** ^b^ **, g/d**								
** Early summer**	16.5	19.9	22.6	22.6	0.96	< 0.01	0.12	0.62
** Late summer**	22.0	25.1	27.4	31.1	1.29	< 0.01	0.84	0.71
**N utilization, %**								
** Early summer**	27.7	34.0	31.0	31.0	1.65	0.39	0.09	0.13
** Late summer**	24.7	27.3	24.1	24.3	0.91	0.30	0.24	0.05

a NC-Negative Control, no supplementation; LS-Light Supplementation, 0.91 kg supplemental extruded DDG cubes/steer provided daily; MS-Medium Supplementation, 1.81 kg supplemental extruded DDG cubes/steer provided daily; HS-High Supplementation, 2.72 kg supplemental extruded DDG cubes/steer provided daily.

b Calculated according to equations from Kohn et al. (2005).

#### Calculated N intake and excretion

Just as BUN concentrations increased as supplementation rate increased (Table 6), increased rate of supplementation proved to linearly increase (*P *≤ 0.01) estimated N intake as well as calculated urinary and fecal N output (*P *≤ 0.05). Nitrogen utilization increased with increasing supplementation rate in Yr 1 during the early summer (Quadratic, *P *= 0.02) but was not impacted by supplement level in the late summer of Yr 1. Nitrogen utilization peaked with the LS treatment in the late summer (Cubic, *P *= 0.05) of Yr 2.

## Discussion

### Forage nutritive quality and biomass

Forage nutritive quality and forage availability have direct implications on response of grazing calves to supplementation. Even though the leaf fraction was separated for forage nutritive quality analysis to attempt to mimic the higher nutritive quality of diets selected by grazing livestock, the forage CP would be considered deficient at all sampling dates for growing beef steers (Table 2 and 3) for all location-years based on NASEM (2016) estimations. Forage CP had a decreasing trend while detergent fiber concentrations (ADF and NDF) increased in both years with corresponding reductions in IVDMD related to advancement in plant maturity as the grazing season progressed. In the early summer, the IVDMD would be sufficient to promote growth rates of grazing steers of 0.8 kg/d or greater, while at the end of the grazing season decreased IVDMD was sufficient to promote ADG of 0.31 kg/d or greater, based on the NASEM (2016) predictions. The estimation of forage CP in the current experiment was problematic based on the performance of NC and calculated N intake (Table 6). Based on NASEM (2016) the forage CP in the diet of NC steers is predicted to be a minimum of 9 to 12% CP for the range of ADG and steer BW observed.

In both years, at all sampling dates the IVDMD: CP ratio was unbalanced (Moore et al. 1999) and is indicative of a ruminal N deficiency. Correction of this ruminal N deficiency with supplementation would likely result in positive associative effects with low supplementation rates by increasing forage digestibility and forage intake if adequate forage availability is present (Moore et al. 1999). The extruded DDGS cubes fed in the current experiment are like those from Adams et al. (2022b) that were 91% total digestible nutrient and 29.9% CP (DM basis). So even though the DDG cubes are exceptionally high in energy their expected IVDMD: CP ratio of 3:1 would ameliorate the imbalance of fermentable organic matter to ruminally available N of the native range forages.

Forage allowance, which is expressed as kg of forage DM per kg of steer BW, is a measure of the relationship between forage availability and the number of animal units on a pasture at a given point in time competing for an optimal diet (Allen et al. 2011). This measure describes the potential for selectivity of grazing by livestock and has been tied closely to potential animal performance (Beck et al. 2013; Rouquette 2016, 2017). The critical point where forage allowance maximizes animal performance is variable for different forage resources (Rouquette 2016) and is different for similar forages under differing seasons or growing conditions (Beck et al. 2013; Rouquette 2016). Using the forage biomass and the average BW throughout the grazing seasons a decline in forage allowance was recognized as the grazing season progressed at both locations. At Klemme, the steers started each grazing season with forage allowances of 25 and 26 in Yr 1 and 2, respectively. By end of summer at Klemme forage allowance fell to 12 in Yr 1 but only declined to 16 in Yr 2. At SPER in Yr 1, forage allowance started at 23 and declined to 13 by the end of the summer grazing season. In Yr 2, stocking rates at Klemme were adjusted to deal with low precipitation during the fall and winter of 2021 and the spring of 2022 going into the second grazing season of this experiment (Fig. 1), while at SPER no adjustments were made to stocking rates. Thus, the forage allowance was much more limited during Yr 2 at SPER. In Yr 2 the forage allowance at SPER started the grazing season at 14 and ended the grazing season at only 6 kg of forage DM/kg of steer BW. This drastic decline in forage availability and forage allowance at SPER in Yr 2 should have had negative influences on selectivity of the diet by grazing cattle (Allen et al. 2011). In a long-term stocking rate experiment reported by Beck et al. (2020), forage allowance at the Klemme site at the end of the summer grazing season declined from 23 kg forage DM/kg steer BW at the lowest stocking rate (4.38 ha/steer) to 9 kg forage DM/kg steer BW at the highest stocking rate (1.83 ha/steer). Even with this large reduction in forage allowance ADG only declined by 0.09 kg/d, which indicates that steers can alter grazing behavior, grazing time, or forage intake rate to offset limited forage availability.

Supplementation appears to have the most consistent response during the late summer, but in the continental climate of the Southern Great Plains region in western Oklahoma this is not necessarily true every summer. Even though late season growth performance increased with supplementation in Yr 2 at SPER, the higher-than-expected performance of NC (0.94 kg/d, Table 5) limited the potential impact of supplementation. We attribute this to the precipitation event in August (Fig. 2) that resulted in regrowth of pastures during the late summer. Anecdotally, steers were observed to spend the preponderance of grazing activity on previously grazed areas that were short but regrowing rapidly. The separation of leaf for nutritive quality analysis does not appear to have adequately captured the nutrient quality of the forages (Table 3) consumed by the grazing steers to support the level of performance observed in unsupplemented NC steers in the late summer of Yr 2 (Table 5).

### Responses to supplementation

Over the entire summer at both research locations, increasing rate of supplementation with the extruded DDG cubes increased steer growth performance, BW at the end of the grazing season, and total BW gain per ha (Tables 4 and 5). However, these experiments indicate that BW gain responses and supplement conversion ratio decreased as supplementation rates increased. This reduction in the marginal improvement in ADG with additional levels of input is a classical response often observed with increasing supplementation rates resulting in the observed reduced supplement conversion ratio (Horn and McCollum 1987).

For both locations and both years, the LS treatment was quite efficient in increasing performance by 0.25 kg/d with an average supplement conversion ratio of 0.28 kg of added gain per kilogram of supplemental DDG cubes. McCollum and Horn (1990) suggested that supplement conversion ratios < 0.25 (> 4 kg of supplement/kg of added BW gain) indicates that energy supply is driving ADG and supplemental nutrients are substituted for nutrients in the basal diet and are thus inefficiently utilized. supplement conversion ratios ≥ 0.33 (≤ 3 kg of supplement/kg of added BW gain) indicate that supplemental nutrients are meeting nutrient deficiencies (McCollum and Horn 1990) and positive associative effects of supplementation are improving forage utilization (Moore et al. 1999).

Supplement responses, and thus supplementation conversation ratio, changed with forage conditions. Consider the case of Klemme in Yr 2 (2022), supplement conversion ratio in the early summer would be considered inefficient at 0.09 and 0.07 for LS and MS, respectively (Horn and McCollum 1987; McCollum and Horn 1990) and in most cases would be considered economically unjustifiable. Meanwhile supplement conversion ratios in the late summer (0.37 and 0.34 for LS and MS, respectively) in the same year would be considered a highly efficient response to supplementation (McCollum and Horn 1990). In a summary of 10 supplementation trials where 0.40 to 0.54 kg/d of 38 to 41% CP supplements were fed for an average of 72 d in the late summer, Beck and Lalman (2021) showed ADG increases of 33% (0.17 kg/d) over unsupplemented controls with supplement conversion ratios averaging 0.36 kg increased ADG/kg supplement (2.77 kg feed per kg added BW gain).

Also, across both years and both locations, the MS treatment increased ADG by an average of 0.35 kg/d with an average supplement conversion ratio of 0.19 kg increased gain for each kg of supplement. The lower supplement conversion ratio with this higher supplementation rate is within the range indicated by McCollum and Horn (1990) where energy supplementation and not protein is the predominant driver in the increase in ADG from supplementation. This supplemental response is similar to observations with lower supplementation rates fed to growing cattle grazing introduced bermudagrass (*Cynodon dactylon*) pastures in more humid regions of the Southeastern US (Gadberry et al. 2010; Beck et al. 2014; Adams et al. 2022a) where forage CP concentrations are generally not limiting to production indicating the impact of forage quality on the efficiency of supplementation response for growing calves supplemented during the growing season.

Across both years at the SPER location, HS increased ADG by 0.41 kg/d with an average supplement conversion ratio of 0.15. Bodyweight gain per hectare increased by 32 to 73% (Tables 4 and 5) as supplementation rate increased. These data show that utilizing the DDG cubes allows a producer to increase productivity of steers grazing summer native range without obvious negative consequences to range conditions and forage availability, this could be due to a forage substitution effect with greater rates of supplementation (Morris et al. 2005; Adams et al. 2022b). This improvement in performance on the same area of land can be beneficial to producers in years that the cattle market incentivizes increased BW gain rather than supplement conversion ratio, such as when values of BW gain are high and feed costs are low.

Adams et al. (2022b) reported that for each 1% of BW increment in supplementation rate, intake of a moderate quality bermudagrass hay decreased by 1.78 kg, or when calculated based on daily supplementation rate each additional kilogram of supplemental DDG cubes reduced forage intake by 0.62 kg. Morris et al. (2005) likewise reported that intake of mixed alfalfa hay and sorghum silage decreased by 0.53 kg for each additional kilograms of distillers’ grains supplement offered while intake of a lower quality forage was not impacted as greatly. When cattle were fed lower nutritive quality bromegrass hay in Morris et al. (2005) there was only a 0.33-kg reduction in forage intake for each additional kilogram of DDG fed. In the current experiment, the LS treatment was 0.27% of average trial BW, while MS a supplementation rate of 0.52% of average BW. At these levels of supplementation, we would expect forage intakes to be decreased by only 0.5 and 1.0 kg/d for LS and MS, respectively, based on Adams et al. (2022b). Cazzuli et al. (2023a) reported that growing heifers fed > 0.7% of BW while grazing stockpiled native Campos grassland during the winter in Uruguay had lower mean forage disappearance from pastures and less time grazing and ruminating than unsupplemented heifers. At SPER, the HS rate was approximately 0.73% of average trial BW. The HS treatment used at the experiments at SPER would thus have potential to offset considerable forage intake (1.25 to 1.33 kg/d based on Adams et al. 2022b) and thus could be used to increase stocking rate of the native range pastures or maintain stocking rates during periods of drought.

Supplement substitution rates of dormant winter range in Uruguay were between 0.3 and 1.1 kg reduction in forage intake per kg of supplement fed replacing 10% to 30% of potential herbage intake (Cazzuli et al. 2023b). Cazzuli et al. (2023b) found that as substitution rates increased there was a reduction in supplement conversion ratio. In the current study, increasing supplementation rates reduced supplement conversion ratio due to reductions in marginal increase in response in ADG as supplementation rate increased. The reduction in supplement conversion ratio in the current experiment was especially notable when supplementation rate increased to HS from MS at the SPER research site, indicating that the higher supplementation rates were likely causing higher substitution rates.

Cazzuli et al. (2023c) found that average supplement conversion ratio was 0.21 kg of added BW gain/kg of supplement fed with average supplementation rates of 1.84 kg/d or 0.84% of BW, which was like our supplementation response with the MS treatment in the current experiments at a lower supplementation rate as a percentage of BW. supplement conversion ratio in Cazzuli et al. (2023c) was positively associated with herbage mass but negatively associated with forage allowance, while associations of supplement conversion ratio were impacted to a lesser extent by supplementation rate. These positive responses of supplement conversion ratio to herbage mass and negative responses of supplement conversion ratio to forage allowance indicate that a fine balance between allowing for grazing diet selectivity and maximizing potential herbage intake must be maintained which may be supportive of the use of higher supplementation rates to drive intensification of production through increasing stocking rates.

### Nitrogen usage and excretion

Ruminal ammonia and BUN concentrations have been shown to be related (Fenderson and Bergen 1976), and the timing of peak BUN levels indicate when rumen ammonia concentrations are peaking (Lewis 1957). Stephenson et al. (2018) indicated that supplementing with a protein meal high in ruminal degradable protein content (cottonseed meal) increased BUN to a greater extent from 6 to 14 h post feeding than what was observed by Adams et al. (2022b) with DDG cubes that have lower rumen degradability. In the current experiment, blood was collected from steers the morning following a 16 h fast suggesting that rumen ammonia levels were maintained following supplementation through N recycling.

Kohn et al. (2005) found a strong linear relationship among BUN concentration and fecal and urinary excretion rate and can be used to effectively predict relative differences in N excretion of similar animals in the same stage of production within a study. Although both rumen degradable and undegradable protein supplements increased plasma urea-N and urinary N compared with control, Huntington et al. (2001) found that increasing rumen undegradable protein of supplemental protein sources from 36% to 65% of CP resulted in lower plasma urea-N but similar urinary N content. Like the calculations in the current experiment, Batista et al. (2016) found that fecal N losses increased linearly with increasing N intake.

Greenquist et al. (2011) estimated N excretion to be equivalent to 112 kg N/ha for grazing steers supplemented with DDG. This surplus N excreted on pastures would be expected to be utilized by growing forages, although urinary N excretion has been estimated to be from 400 kg N/ha (Haynes and Williams 1993) to up to 1000 kg N/ha (Dijkstra et al. 2013) in the urine patch. It was estimated that only 20% of the N ingested by cattle on low nutritive quality native range pastures is excreted as urinary N while up to 75% is excreted as urinary N by livestock grazing high quality temperate pastures (Russelle 1992). Due to its high concentration in localized spots, urinary N uptake by plants can often be only a small fraction of the excreted N, with losses ranging from 15% in subtropical grasslands to 25% in temperate grasslands and may be up to 90% in semiarid regions (Russelle 1992). Fecal N on the contrary is primarily organically bound and slowly degraded into forms usable by plants (Russelle 1992).

A review by Dijkstra et al. (2013) concluded that reducing urinary N output or urinary N concentration and shifting N excretion to increase fecal N excretion is a mechanism to improve N cycling and reduce N losses. Wickersham et al. (2008) found that as supplementation rates of DIP increased to steers fed low-protein forage diets both fecal and urinary N excretion increased by 80 and 68%, respectively, but with very little shift in the ratio of fecal N excretion to urinary N excretion remaining very close to a 60:40 ratio at all levels. This observation is opposed to the findings of Marini and Van Amburgh (2003), who found that increasing the dietary N intake of heifers fed isoenergetic total mixed diets resulted in increased urinary N excretion from 32% urinary N excretion with the 9.1% CP diet to 71% urinary N excretion with the 21.3% CP diet. In the current experiment, the calculated urinary N excretion was approximately 60% of total excretion with very little change in the fecal: urinary N excretion ratio across the range of supplementation rates.

Forage growth of the sward area adjacent to fecal patches has been estimated to affect areas 5 to 6-times larger than the dung patch itself and may last up to 2 yr while in urine patches N volatilization losses occur more rapidly during the first 24-h and then decline over time (Haynes and Williams 1993). Pasture growth responses of 7-fold have been observed in the urine patch but the increase with this N source last only 2 to 3 months. Urine spots and fecal pats are usually spatially separated in pastures except near watering or bedding areas leading to variability of excreted N distribution (Russelle 1992).

## Conclusions

Gunter (2019) showed that increasing supplementation rates of steers on dormant native range were most beneficial at higher stocking rates compared with moderate stocking rates. This can be beneficial to producers, allowing options to utilize low-quality grazing forages more effectively to improve animal performance at higher stocking rates. The improvement in performance with increasing supplementation rate in the current experiment can be seen in the improved animal BW gain per hectare, thus increasing the potential for economic returns on the same area of land (Beck et al. 2020). Although supplementation can alleviate potential nutritional deficiencies, proper grazing management is necessary to maintain native grazing land for future use. Rainfall is essential to forage production on native range; this has become more evident to producers in recent years with drought conditions sustaining over multiple years (Beck and Beck 2025). At both locations, rainfall was near the long-term average in 1 yr and below the long-term average rainfall (Figs. 1 and 2) in the second, which impacted forage biomass as the grazing season progressed (Tables 2 and 3). Producers could potentially look to supplementation strategies to increase forage utilization efficacy of forage that is available in years with normal rainfall and a higher supplementation rate in drought years to offset forage production deficits.

Though blood urea N values are elevated with increasing rate of supplementation, excreted N can play a beneficial role to the N cycle in the soil for future forage growth (Wallis et al. 2023). Wallis et al. (2023) stated that providing supplementation of nutrients to grazing cattle rather than through practices such as fertilizing pastures, can increase sustainability of a grazing operation. These sustainability factors include improved animal performance, N use efficiency and mitigation of nitrogenous emissions while optimizing nutrient cycling in the animals as well as in the environment.

## Data Availability

The dataset supporting the findings of this study, *“Effects of increasing supplementation rates of extruded distillers’ grain cubes on stocker steers production grazing native range in Bessie, OK and Fort Supply, OK during the summer months in 2021 and 2022”* (Gunter, S.; Grisby, Z.; Beck, P.; Friend, E. M., 2025) is publicly available in the Ag Data Commons repository at https://doi.org/10.15482/USDA.ADC/29610539, under DOI: 10.15482/USDA.ADC/29610539.
